# Purple reign: the outsized impact and potential of Chromobacterium on quorum sensing research

**DOI:** 10.1099/mic.0.001765

**Published:** 2026-11-05

**Authors:** Eryk J. Yarkosky, Samantha W. Miranda, Helen E. Blackwell, Josephine R. Chandler

**Affiliations:** 1 Department of Molecular Biosciences, University of Kansas, 1200 Sunnyside Avenue, Lawrence, KS 66045, USA; 2 Center for Integrated Solutions for Infectious Diseases, Broad Institute of MIT and Harvard, 415 Main Street, Cambridge, MA 02142, USA; 3 Department of Chemistry, University of Wisconsin–Madison, 1101 University Avenue, Madison, WI 53706, USA

**Keywords:** acyl-homoserine lactones, *Chromobacterium*, quorum sensing, violacein

## Abstract

Members of the *Chromobacterium* genus – instantly recognizable for their vivid purple pigment – have played a disproportionately influential role in shaping quorum sensing research. The discovery that violacein production in the model species *Chromobacterium subtsugae* is governed by an *N*-acyl l-homoserine lactone (AHL)-based LuxI/LuxR signalling system ignited a wave of breakthroughs in the field. With its genetic simplicity and visually striking phenotype, *Chromobacterium* emerged as a powerful and accessible model, enabling sensitive detection of AHLs across diverse bacteria and opening the door to fundamental insights into signalling promiscuity, interspecies communication and the molecular logic of quorum sensing pathways. In this review, we highlight both the foundational discoveries and ongoing contributions of the *Chromobacterium* genus, arguing that it has been central not only to the origins of AHL-based quorum sensing research but also to the field’s expansion towards increasingly sophisticated, community-level perspectives that continue to define its trajectory today – and into the future.

## Introduction

The *Chromobacterium* genus, characterized by its production of a striking purple pigment, has played a pivotal role in shaping our current understanding of bacterial cell–cell signalling and has arguably helped define the field of quorum sensing. Members of this genus, and particularly a widely used laboratory strain of *Chromobacterium subtsugae* (historically classified as *C. violaceum*), were critical for expanding quorum sensing research and making the field accessible to scientists from a variety of disciplines and places. Despite this impact, the central role of *Chromobacterium* in the development of quorum sensing research is often underrecognized.

In this review, we highlight the many ways in which the purple-pigmented *Chromobacterium* has shaped the understanding of quorum sensing over the past 30 years, becoming one of the arguably most well-known and widely used bacteria in the quorum sensing field. We showcase the central role of the *Chromobacterium* genus from the early development of a *Chromobacterium*-based biosensor of quorum sensing signals to the discovery of synthetic quorum sensing inhibitors (QSIs) to its emerging use as a model for studying microbial interactions. Together, these studies underscore both the origins and continued development of the quorum sensing field.

## *Chromobacterium* for detecting *N*-acyl l-homoserine lactones and other quorum sensing modulators

The earliest strain of *Chromobacterium* (*C. bergonzini*, now named *C. violaceum*) was initially reported in 1881 [1–3]. This strain and many others isolated since have been characterized by their production of a bright violet pigment, the result of the biosynthesis of an antimicrobial natural product called violacein [4, 5]. Violacein is of interest to biomedical research for its anti-tumour activity, anti-parasitic activity and antibacterial activity, as well as in industrial settings as a brilliant dye for textile and cosmetic products. For this reason, violacein biosynthesis and production were the focus of early research on those members that produce it [6, 7]. Some *Chromobacterium* species are also human pathogens that cause rare but severe disease ranging from cellulitis to septicaemia in compromised individuals, particularly in tropical and subtropical regions, prompting later studies of virulence mechanisms [8, 9].

In 1997, a study by McClean *et al*. [10] linked violacein production with LuxI/LuxR-type quorum sensing. Quorum sensing is found in many *Proteobacteria* and relies on the production and detection of *N*-acyl l-homoserine lactone (AHL) signals. AHLs are synthesized intracellularly via LuxI-type synthases, typically diffuse in and out of the cell, and once they reach a threshold concentration in the local environment (and in the cell), they bind and activate cytoplasmic LuxR-type transcription factors. The McClean study [10] identified a transposon insertion mutant of *C. subtsugae* CV017 (referred to at the time as *C. violaceum*) that was non-pigmented, suggesting that violacein production was genetically disrupted. The transposon insertion was mapped to an AHL signal synthase gene, which the authors called *cviI*. The region around *cviI* was cloned and sequenced, and an adjacent AHL receptor gene was identified, called *cviR*. CviI was shown to synthesize an AHL, *N-*hexanoyl l-homoserine lactone (C6-HSL). The results demonstrated that violacein production required the CviI/CviR circuit, and adding exogenous C6-HSL to the original CviI-disrupted transposon mutant (called strain CV026) was sufficient to restore purple pigmentation (Fig. 1a, b). Other AHLs also induced violacein production in CV026, such as *N*-butanoyl l-homoserine lactone and *N*-octanoyl l-homoserine lactone (C4-HSL and C8-HSL, respectively), suggesting that the CviR receptor was relatively promiscuous and could potentially sense and respond to AHLs from other species. Interestingly, AHLs with longer-chain acyl groups such as C10-HSL and C12-HSL act as CviR antagonists in the presence of C6-HSL and repress violacein production [10, 11].

**Fig. 1.**
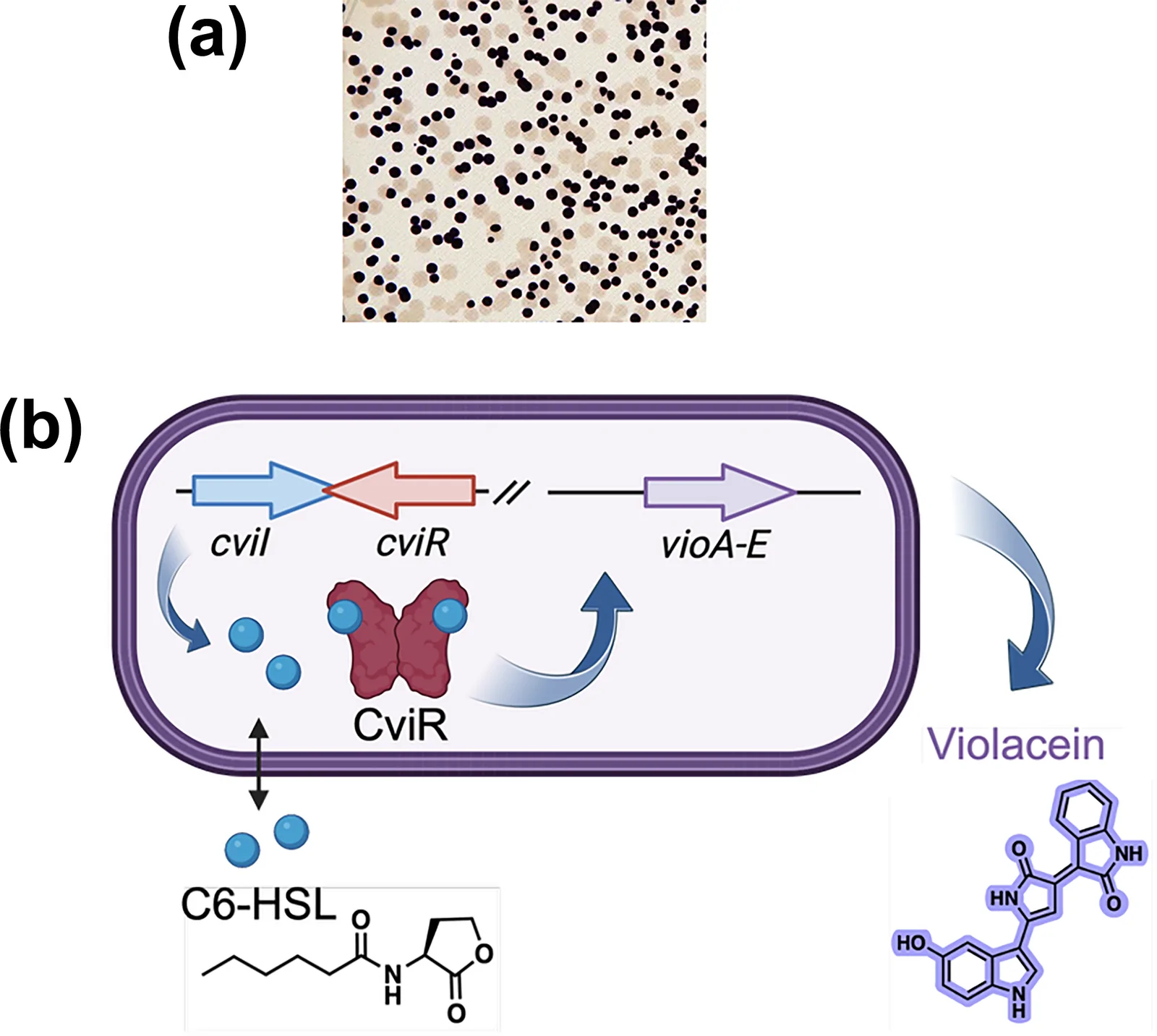
(a) Spread plate on agar of isolated colonies of a mixture of two strains of *Chromobacterium subtsugae*. Purple colonies are strain CV017, coloration is due to production of the purple pigment violacein. Cream colonies are strain CV026, loss of coloration is due to disruption of the violacein-activating CviI/CviR AHL quorum-sensing circuit. (b) Schematic of the *C. subtsugae* CviI/CviR quorum-sensing circuit. Blue circles indicate the native ligand for CviR, C6-HSL. C6-HSL-bound CviR activates transcription of the *vioABCDE* genes coding for biosynthesis of the purple pigment, violacein.

Of note, the CV017 strain has a gene disruption in CV_1055 (later named *vioS*) [10–12]. This disruption enhances production of violacein and is responsible for the hallmark purple colour of this strain that was helpful in identifying violacein-defective mutants in the above screen; the disruption is absent in the parent of CV017 (ATCC31532), which is a much less vivid purple, closer to a light lavender colour. Although the mechanism of VioS is not well understood, it represses CviR-dependent activation of transcription of the first violacein biosynthetic gene (*vioA*) in a heterologous *Escherichia coli* host and also represses two other CviR-dependent phenotypes (chitinolytic and protease activity) [11], supporting a model of VioS function by regulating CviR either by competing for binding at the target gene promoter or through a direct protein-protein interaction analogous to the PqsE regulator of RhlR in *Pseudomonas aeruginosa* [13, 14]. A recent yeast two-hybrid experiment provided evidence that VioS interacts directly with CviR [15], supporting the latter model of a protein-protein interaction mechanism of regulation by VioS.

The McClean *et al*. study prompted the widespread adoption of CV026 as a biological sensor for the detection of AHLs. This approach provides a low-cost and easily accessible strategy for screening AHLs because violacein can be detected using standard absorbance assays [16]. It also has a relatively broad range of detection (from 4- to 8-carbon chains). Although no single biosensor can detect the full range of AHLs, CV026 is informative when used alongside other biosensor strains such as *Rhodobacter capsulatus* ALS1(pYP) for detecting C14-C16 AHLs [17] and *Agrobacterium tumefaciens* KYC55(pJZ372)(pJZ384)(pJZ410) for detecting C6-C10 and some C12 AHLs [18] (for further discussion of biosensor strains for AHL detection, see [19, 20]). Samples can also be separated based on retention behaviour using thin-layer or liquid chromatography [21]. This step is often taken prior to AHL detection with biosensor strains, and the separated AHL activities are compared with that of commercially available AHL standards to putatively identify unknown AHLs [20]. Such separation can also be useful to resolve samples that contain multiple AHLs, as AHLs can have overlapping or antagonistic activities in biosensor assays. Exact structural determination of an AHL is carried out by mass spectrometry that can be coupled with NMR to resolve double bond positions, stereochemistry or other unusual functional groups [20, 22].

The use of the CV026 biosensor strain led to an explosion of applications in the quorum sensing field and, together with other broadly selective biosensors (e.g., [23]), was a key driver in establishing the distribution and diversity of AHLs in many proteobacterial species (e.g., [24–28]). Access to such a straightforward method to assay quorum sensing activity prompted the use of CV026 and related strains in screens for chemical modulators of quorum sensing, with a special emphasis on QSIs (for selected references, see [29–41]), ranging from highly focused studies to more high-throughput screening campaigns [42]. For example, a CviR reporter was central to identifying the well-known synthetic antagonist chlorolactone (CL) [43], which was subsequently shown to directly inhibit CviR and provided the first structural insights into LuxR-type protein antagonism (see below). Numerous other synthetic and naturally occurring modulators of violacein production have been reported to date (acting via CviR and likely other pathways), with the search term ‘*Chromobacterium*’ and ‘assay’ receiving over 750 literature citations in the past 30 years (PubMed search April 2026), work advanced by the initial discovery of CV026.

Today, CV026 is one of the most widely used strains in the AHL quorum sensing field. Its ubiquity is further underscored by the routine use of this strain in classroom settings, often based on the straightforward approaches described by McClean *et al*. [10]. However, the broad selectivity that makes CV026 and similar biosensors so useful is also limiting and potentially problematic in that they cannot definitively identify the specific AHL being produced without additional analytical approaches. As a result, reliance on these biosensors alone has contributed to AHL misidentification and confusion regarding signal specificity in many systems. Recent efforts to more rigorously link individual AHLs to their cognate synthases based on the strength of experimental evidence have helped bring greater clarity to this issue [44].

## Other studies of quorum sensing in *Chromobacterium*

Our understanding of quorum sensing in *Chromobacterium* has primarily come from studies of *C. violaceum* and *C. subtsugae*. These species have been used to uncover mechanisms of the AHL synthase and receptors and more generally as a model to study the role of quorum sensing in bacterial interactions, each of which will be described in this section.

### Quorum-sensing circuits

In *C. subtsugae*, the CviI/CviR circuit produces and responds best to C6-HSL [10]. In *C. violaceum*, this circuit (referred to here as CviI’/CviR’) appears to produce and respond best to *N-*(3-hydroxydecanoyl)-l-acylhomoserine lactone (3-OH-C10-HSL) [45]. Receptors of both species are relatively promiscuous, with each detecting a different subset of AHLs [10, 45]. Further, the sensitivity to particular AHLs can be altered by only single or double amino acid substitutions in each receptor protein [43]. Studies of CviR’ showed that as with most other AHL receptors, when bound to AHL, it recognizes a well-conserved palindromic DNA sequence in the promoter region of its target genes that is more generally referred to as the *lux* box [46]. CviR’ and CviR can activate dozens of genes, including their cognate signal synthases, *cviI* [6–9]. The production of antimicrobials, such as violacein and hydrogen cyanide, is also controlled by these circuits [10, 47].

The structure of full-length and partial-length CviR has been solved at ~2.5–2.7 Å resolution in complex with antagonist and agonist (C6-HSL) ligands, respectively [44]. CviR has the canonical architecture of other AHL receptors, with an N-terminal ligand-binding domain and a C-terminal helix-turn-helix DNA-binding domain. However, comparisons with the solved structure of the related LuxR homologue – TraR of *A. tumefaciens* [48] – bound to both native AHL and DNA revealed an unexpected mechanism for discrimination of the native AHL ligand and non-native antagonists that is illustrated in Fig. 2(a). In a complex with the native AHL, CviR is predicted to adopt a confirmation compatible with dimerization and DNA binding, positioning the DNA-binding domains appropriately to interact with the conserved recognition sequence in the DNA of the target gene promoter. In contrast, when bound to a synthetic antagonist (e.g., CL) or a non-native AHL that causes CviR inhibition, the CviR homodimer appears to undergo a major conformational rearrangement in which the DNA-binding domains are misaligned with the DNA recognition sequence, which was proposed to be key for antagonism. The results also suggest that although CviR can bind to a range of AHLs accounting for its promiscuity in AHL specificity, small differences in the AHL side chain can have major consequences for the protein conformation and DNA-binding ability. These studies provided one of the first structural explanations for how LuxR-type receptors discriminate native AHLs from other ligands, such as antagonists, and offered a mechanistic basis for the promiscuity of CviR. More broadly, the studies provide a mechanistic rationale based on ligand-dependent conformational switching for transcriptional activation and inactivation in this family of regulators. Determining if this mechanism is shared across other LuxR-type receptors or is specific to CviR remains an open question.

**Fig. 2.**
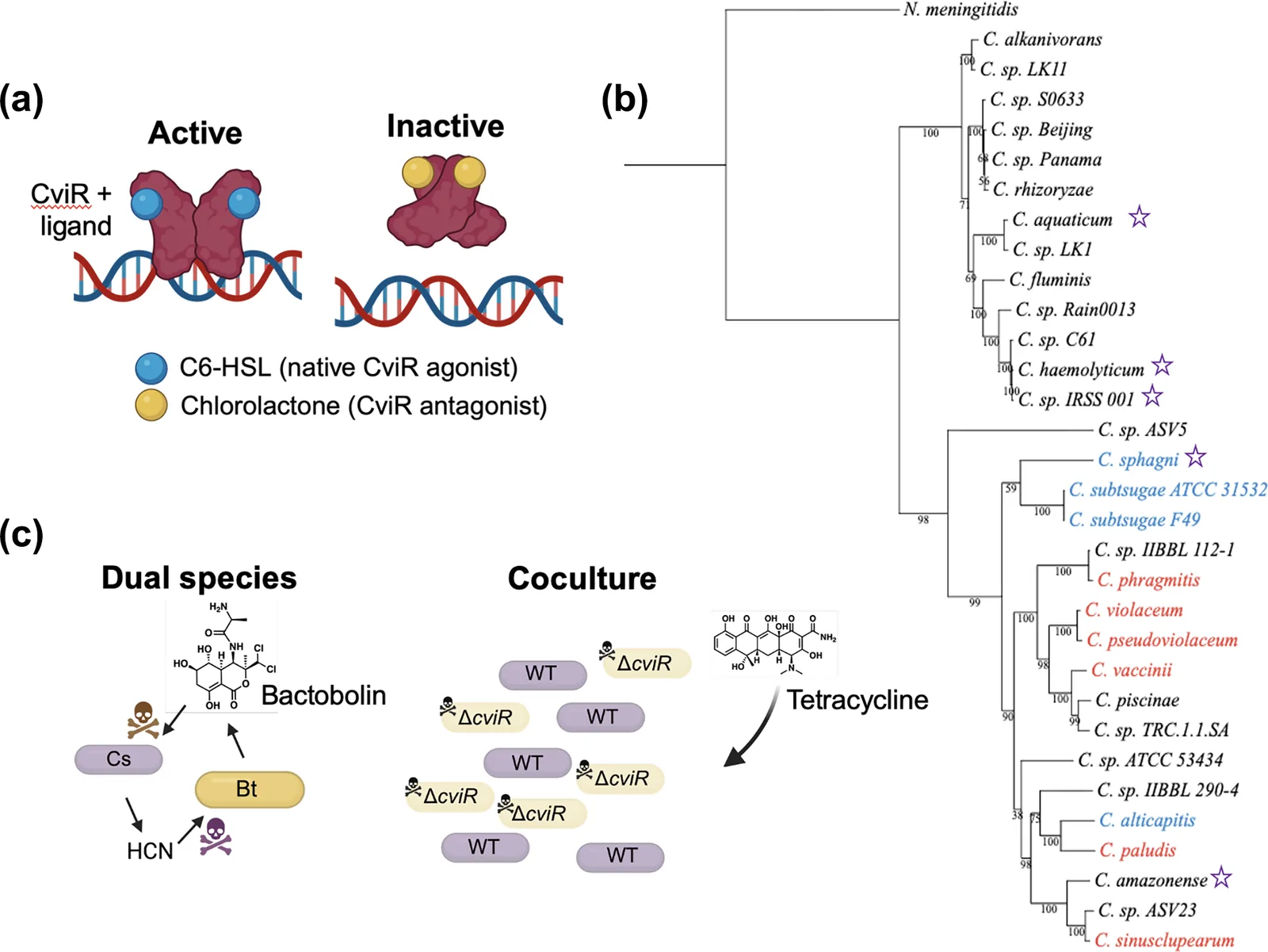
(a) Proposed model of ligand-dependent conformational switching based on CviR structures. When bound to the native ligand C6-HSL (blue circles), CviR forms an active conformation that can bind a conserved DNA sequence in the promoter of the target gene. When bound to the antagonist CL (yellow circles), CviR forms a conformation that is unable to bind DNA. (b) Maximum likelihood phylogenetic tree of *Chromobacterium* species and an outgroup (*Neisseria meningitidis*). The tree was generated using the Bacterial and Viral Bioinformatics Resource Center (BV-BRC) [50]; 100 conserved genes were selected using the BV-BRC cross-genera protein families (PGFams) tool [52], and the tree was generated using RAxML [53]. In the tree, species with AHL synthases similar to that of *C. subtsugae* are in blue text, those similar to *C. violaceum* are in red, and AHL synthases distinct from these two groups are in black; similarities are based on a network similarity analysis with an alignment score of 85 [54-57] using the AHL synthases of all publicly available *Chromobacterium* strains [41] and characterized AHL synthases from the RefAHL database [33]. (c) Laboratory models of interbacterial interactions with *C. subtsugae*. Left, dual-species with *C. subtsugae* (Cs) and *Burkholderia thailandensis* (Bt). The *C. subtsugae* CviR-CviI quorum sensing system activates production of hydrogen cyanide (HCN), which increases its competitiveness by killing *B. thailandensis*. *B. thailandensis* uses an AHL quorum sensing system (BtaI2/BtaR2) to activate production of bactobolin, which increases its competitiveness by killing *C. subtsugae*. Right, cocultures of *C. subtsugae* WT and ∆*cviR* strain. The exogenous addition of tetracycline selectively kills ∆*cviR*, altering population dynamics and promoting the maintenance of quorum sensing.

### Distribution of LuxI/LuxR quorum sensing circuits in the Chromobacterium genus

Although there have been relatively few experimental studies of Chromobacterium LuxI/LuxR circuits outside of *C. violaceum* and *C. subtsugae* [49], our analysis of publicly available genomes [50–52] indicated that all members of the genus *Chromobacterium* possess at least one AHL circuit. While most species have a single circuit, members of *C. haemolyticum*, *C. sphagni*, *C. amazonense* and *C. aquaticum* each encode two circuits (Fig. 2b, indicated by stars). Sequence similarity network analysis of LuxI-type synthases from *Chromobacterium* and other characterized quorum sensing systems [44, 53–56] shows that these second circuits are more closely related to one another than to other known LuxI-type synthases, suggesting that they may represent a distinct functional group. Notably, the species with two circuits do not appear closely related in a phylogenetic tree of representative *Chromobacterium* species (Fig. 2b), raising the possibility, among others, that this second circuit originated in a common ancestor of the genus and was subsequently retained or lost in different lineages. Among the other LuxI-type synthases in the genus, only a few share similarity with those of *C. violaceum* or *C. subtsugae* (Fig. 2b, indicated by red or blue text, respectively); this similarity suggests that this small set may produce 3-OH-C10-HSL and C6-HSL. The other putative synthases are dissimilar from any known LuxI-type synthases [44], indicating the potential for additional signal diversity that remains unexplored. Looking forward, this tapestry of LuxI/LuxR circuits in the *Chromobacterium* genus provides an excellent system for studying the acquisition and loss of multiple quorum sensing systems and the evolution of LuxR-type receptor specificity.

### Quorum sensing in microbial communities

*C. subtsugae* is an emerging model for studies to understand quorum sensing in the context of interbacterial interactions. Its genetic tractability, simple growth requirements and the promiscuity of its CviR receptor make this organism especially well-suited for these types of studies, which often require growth with other strains or species. For example, studies of cocultures with *C. subtsugae* and another soil bacterium, *B. thailandensis*, showed that these two species compete using quorum sensing controlled antimicrobials; these are bactobolin antibiotics produced by *B. thailandensis* and the hydrogen cyanide antimicrobial produced by *C. subtsugae* [57] (Fig. 2c). In addition, *C. subtsugae* can respond to *B. thailandensis* AHLs using the CviR receptor [57], illustrating how AHL-dependent crosstalk, often referred to as ‘eavesdropping’, is a potential advantage of LuxR-type receptors with relaxed AHL specificity during interspecies competition. In the *C. subtsugae*–*B. thailandensis* model, AHL-dependent crosstalk caused CviR in *C. subtsugae* to activate hydrogen cyanide production, which subsequently killed *B. thailandensis*. These results are exciting as they suggest that AHL-dependent crosstalk could provide a possible mechanism for bacteria to sense and respond to their neighbours and shift towards more competitive behaviour strategies.

Beyond AHL crosstalk, studies of *C. subtsugae* also illustrate how quorum sensing integrates diverse environmental cues; in one study, antibiotics (i.e., produced by other species) were shown to activate the CviI/CviR system, enabling a chemically induced ‘counterpunch’ that increased competitiveness with other species [58]. Research on *C. subtsugae* quorum sensing also contributed to the current understanding of bacterial cooperation [59]. In *C. subtsugae*, CviR controls production of an extracellular protease, which is required for nutrient acquisition during growth on protein substrates (such as casein). As has been shown in other species [60–63], protease production in *C. subtsugae* benefits all members of the population and can be exploited by protease-defective cheaters that can eventually drive the population to collapse; one class of cheater is quorum sensing receptor (CviR)-deficient mutants. In *C. subtsugae*, CviR deficiency also increased sensitivity to certain antibiotics [59, 64]. Adding antibiotics to casein-grown cultures prevented the proliferation of CviR-deficient cheaters and averted population collapse. Thus, in addition to amplifying ‘offensive’ strategies during competition, such as antibiotic production, bacteria can also use quorum sensing to stabilize cooperation. Together, these studies underscore the versatility of *C. subtsugae* as a model for probing eavesdropping, competitive responses and the maintenance of cooperation in complex microbial communities.

## The future: what questions come next?

*C. subtsugae* and other members of the *Chromobacterium* genus have been an important cornerstone of quorum sensing research since the beginnings of the field. The biosensor strain has been widely used for the identification of novel quorum sensing bacterial isolates and natural and synthetic QSIs. Members of this genus have also advanced our understanding of the mechanisms by which LuxR-type receptors distinguish and respond to different ligands through conformational changes. The *C. subtsugae* AHL quorum sensing system has also been critical for foundational studies demonstrating the important role of AHLs during interspecies competition through eavesdropping and coordination of competitive strategies.

Looking to the future, the variation in native AHL ligands produced by members of the *Chromobacterium* genus, paired with emerging models of receptor-ligand interactions from structural and biochemical studies of LuxR-type receptors [22, 43, 65–67], could aid in the continued advancement of how we conceptualize LuxR-type receptor selectivity and specificity, and the evolution of quorum sensing systems. We also anticipate the ongoing development of computational tools that can be used to make accurate predictions of LuxI-type synthase and LuxR-type receptor functionalities. Such advances would mark a shift in how quorum sensing systems are studied and expand understanding of evolved differences in receptor specificity/selectivity and the role of these signalling systems in interspecies interactions. Within this emerging landscape, we propose that the *Chromobacterium* genus stands out as an especially powerful and versatile model system, uniquely positioned to drive the next generation of discovery in quorum sensing biology.

## Abbreviations

AHL: *N*-acyl l-homoserine lactone
C6-HSL: *N*-hexanoyl l-homoserine lactone
CL: chlorolactone
3-OH-C10-HSL: *N*-(3-hydroxydecanoyl)-l-acylhomoserine lactone
QSIs: quorum sensing inhibitors
